# A Rare Case of Nonsyndromic Generalized Radiculomegaly with a Literature Review

**DOI:** 10.1155/2020/3974829

**Published:** 2020-03-26

**Authors:** Mohammad Al-Obaida

**Affiliations:** Department of Restorative Dental Sciences, King Saud University, Riyadh, Saudi Arabia

## Abstract

Radiculomegaly is a rare condition involving elongated tooth roots. This condition has significant clinical implications and has been associated with syndromes such as oculofaciocardiodental syndrome. However, only a few nonsyndromic cases of radiculomegaly have been reported. Here, we report a case of nonsyndromic radiculomegaly in a Saudi individual. A subsequent review of the literature suggests endodontic management modalities for individuals with the dental findings demonstrated in the present case.

## 1. Introduction

Tooth development is a complex physiological process which involves bud, cap, and bell stages, as well as tooth root development and tooth eruption. After a crown is nearly completed, the root begins to develop according to guidance provided by the double layer of the epithelial sheath referred to as “Hertwig's epithelial root sheath” [1]. The most common root malformations in humans arise from either developmental disorders of the root alone or disorders of radicular development as part of general tooth dysplasia [2]. However, there is limited information regarding mechanisms of root morphogenesis and elongation [3].

Abnormal tooth morphology and number, including hyperdontia, hypodontia, oligodontia, multiple roots, and taurodontia, are obvious dental anomalies [4]. Among these anomalies is a rare congenital condition called, “radiculomegaly,” which involves a pronounced elongation of dental roots (either in isolation or affecting multiple teeth). This rare condition has seldom been reported in the literature. The first reports of radiculomegaly [5–7] described it as a characteristic of the X-linked rare congenital syndrome, oculofaciocardiodental syndrome (OFCD), which only affects females. However, radiculomegaly was later reported as a characteristic feature of males affected by Klinefelter syndrome [3, 8]. To date, only a few nonsyndromic cases of radiculomegaly have been reported [9, 10]. Here, we report a case of nonsyndromic radiculomegaly in a Saudi individual and then discuss the aspects of this case in relation to previous studies (Table 1).

## 2. Case Report

### 2.1. Patient History

A 31-year-old Saudi male patient presented to a restorative clinic at the College of Dentistry, King Saud University, Saudi Arabia. The patient was seeking permanent restoration of an endodontically treated tooth on the left side of his lower jaw which had undergone significant decay many years prior.

The dental history of the patient included extraction of a mandibular left molar, multiple restorations, and a root canal treatment. The patient stated that he underwent a surgical procedure to the gingiva to help his anterior permanent teeth erupt during childhood. Medical history revealed no significant reports. The family history for the patient also revealed no evidence of any medical condition.

### 2.2. Physical Examination

**Figure 1:**
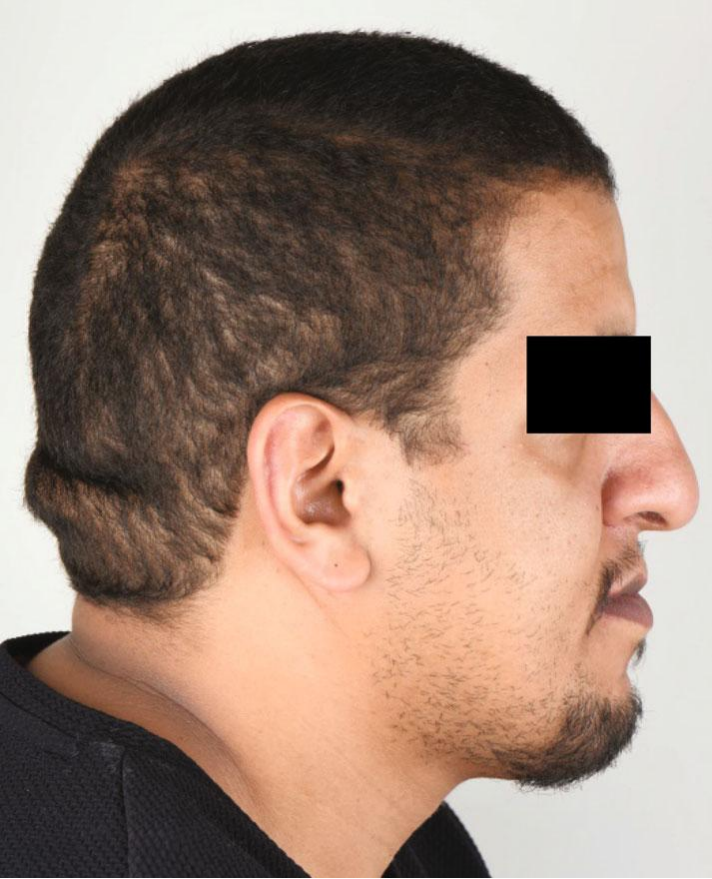
A profile view of the patient's mandibular protrusion.

**Figure 2:**
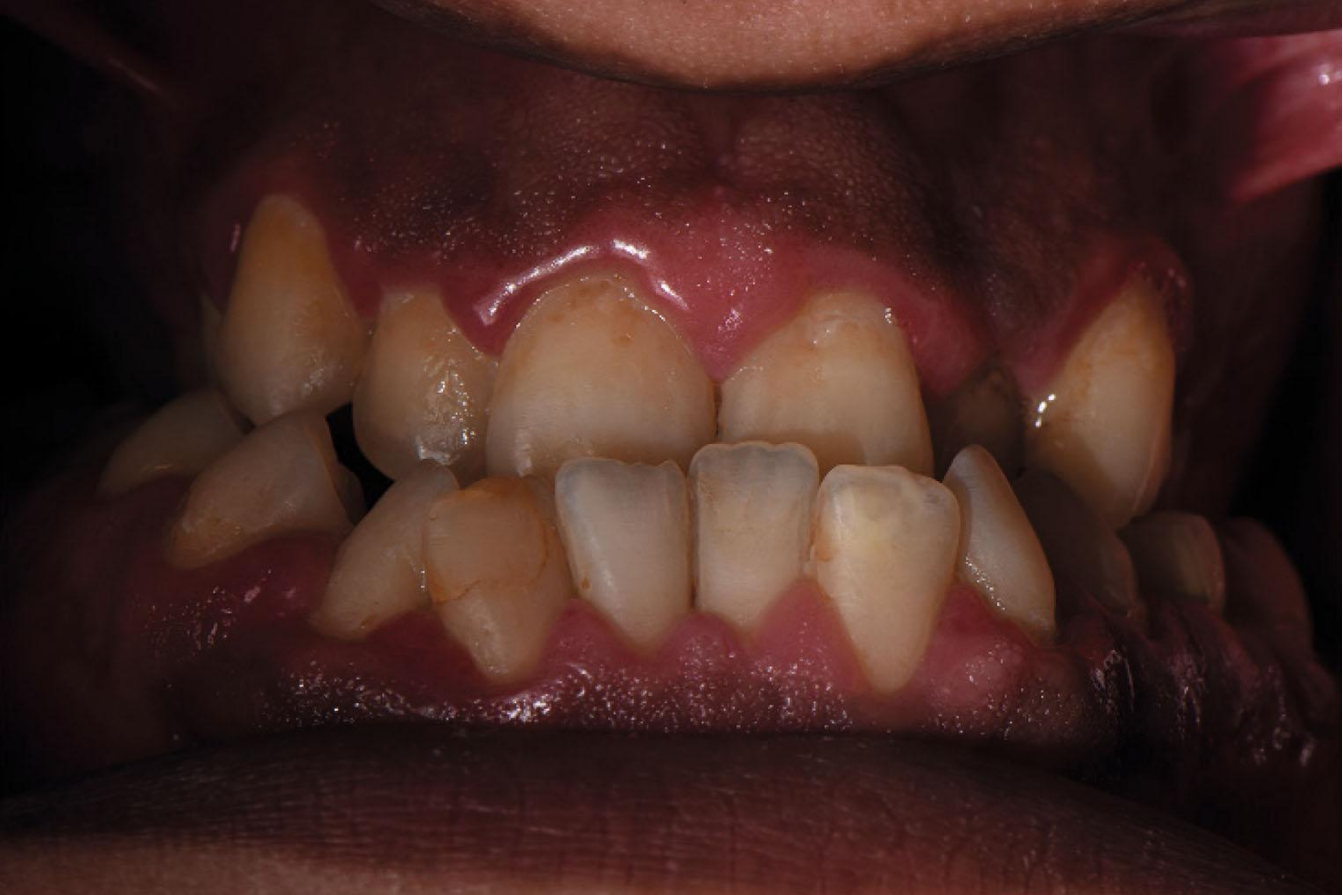
An intraoral anterior view of the patient's anterior cross bite.

**Figure 3:**
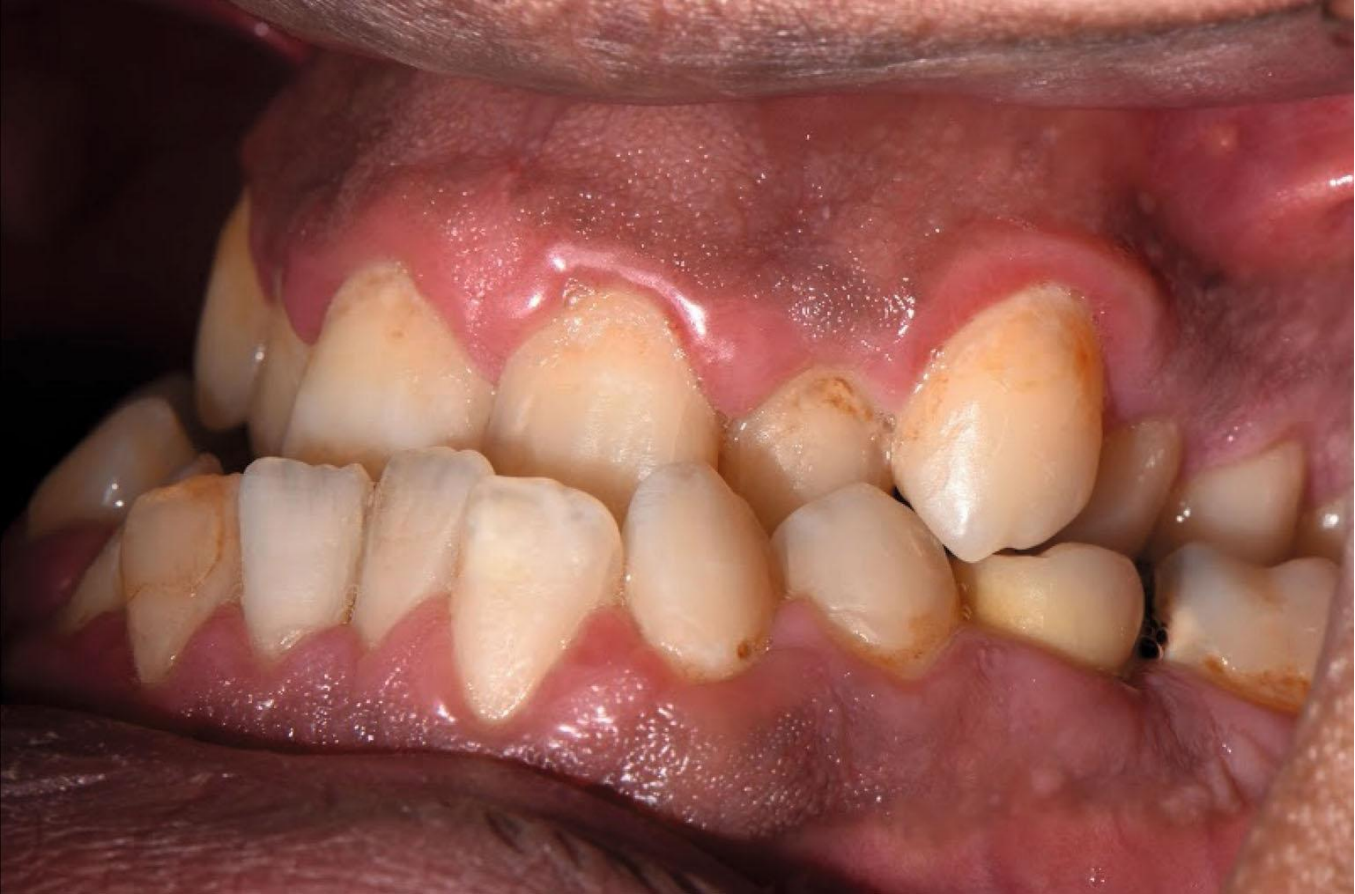
An intraoral lateral view of the patient's posterior cross bite.

**Figure 4:**
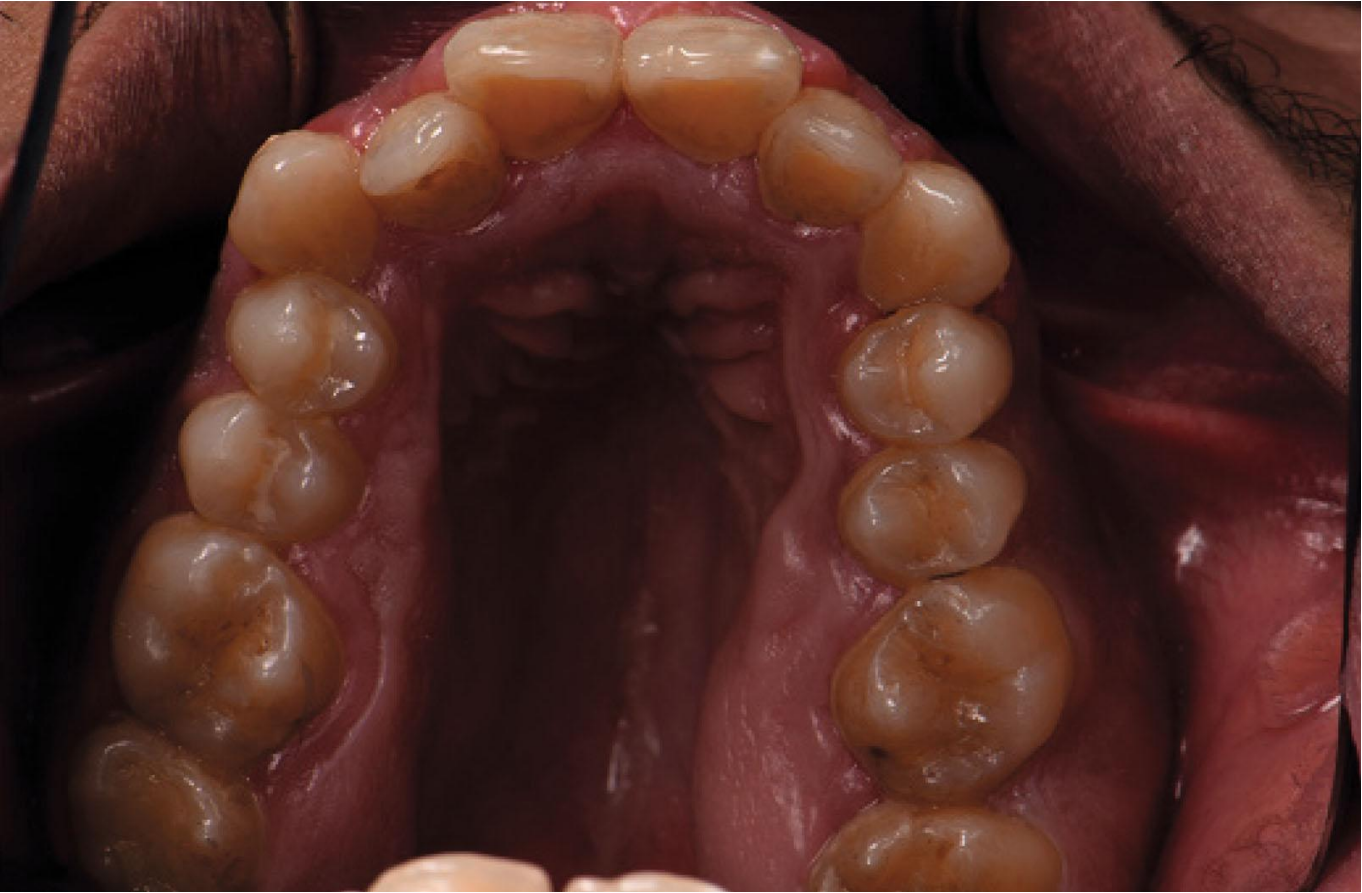
A view of the deep V-shaped maxillary vault of the patient. Bone enlargement in the molar area is also observed.

Height and weight recorded for the patient were 185 cm and 114 kg, respectively. A head and neck extraoral examination revealed a mandibular protrusion leading to a straight facial profile (Figure 1) and tight lips upon closure with a thick lower lip. An intraoral examination showed racial melanin pigmentation, especially on attached gingiva, with anterior and bilateral cross bites (Figures 2 and 3). Generalized gingivitis with bad oral hygiene and heavy plaque accumulation were observed in the crowded anterior segments. Areas of deep pockets and bleeding were also noted. Tooth #32 presented hypomineralization, while a very deep vault in the hard palate and bony enlargement of the posterior areas of the maxillary arch were also observed (Figure 4). Crowded teeth characterize both of the patient's arches since the patient has a full set of teeth and narrow upper and lower arches.

### 2.3. Radiographic Examination

**Figure 5:**
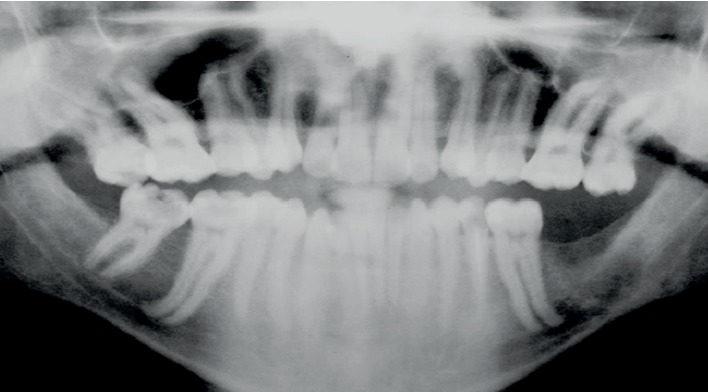
A panoramic radiograph showing the elongated roots of the teeth.

**Figure 6:**
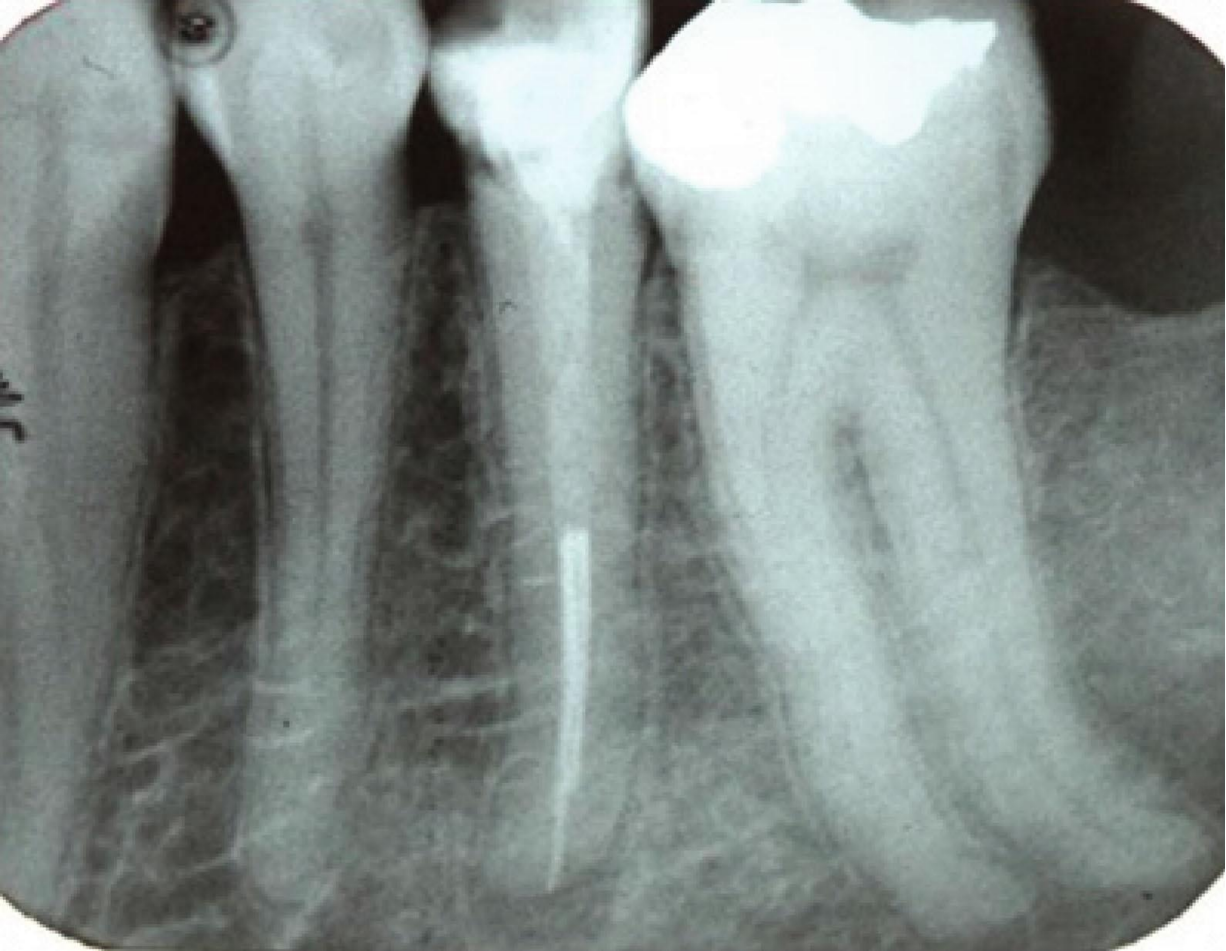
PA X-ray of the long root of the teeth.

**Figure 7:**
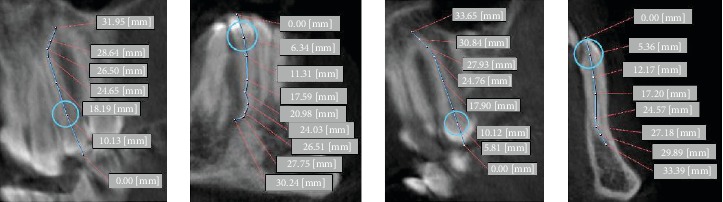
CBCT view of some teeth.

A panoramic view of the patient's teeth show long roots (radiculomegaly) for most of the teeth, especially for the molar teeth (Figure 5). Periapical radiographs also show unusually long roots (Figure 6). Cone beam computed tomography (CBCT) software was used to measure the length of the patient's entire set of teeth. Measurements were made from the apex to the tip of the cusps in a slice orientation manner (Figure 7).

## 3. Discussion

Radiculomegaly, a condition which manifests as elongated dental roots (whether isolated or generalized), is rare. Radiculomegaly was first mentioned as an entity in 1980 [5], and then subsequently in 1986 and 1990 [6, 7]. In 1990, Marashi and Gorlin further observed a potential association between radiculomegaly and cataracts. Based on their observations, they proposed the likelihood of a syndrome [11]. In 1993, Wilkie et al. hypothesized that a combination of eye, facial, heart, and teeth abnormalities represent characteristics of OFCD. Support for this hypothesis was subsequently provided by Gorlin et al. in 1996.

In the present case, an association between radiculomegaly and OFCD was excluded since OFCD is an X-chromosome linked disease and is fatal in males [4, 10, 12]. Our patient also did not exhibit any eye or heart problems. Similarly, Klinefelter syndrome was excluded since males with Klinefelter syndrome often manifest clinical signs and symptoms which affect their physical and intellectual development (e.g., infertility, primary testicular insufficiency, breast enlargement (gynecomastia)). Other manifestations can include abnormal fusion of certain bones in the forearm (radioulnar synostosis), curved pinky fingers (fifth finger clinodactyly), and flat feet (pes planus). Furthermore, individuals affected by Klinefelter syndrome tend to experience anxiety, depression, impaired social skills, behavioral problems (e.g., emotional immaturity, impulsivity, attention-deficit/hyperactivity disorder), and limited problem-solving skills (executive functioning). Approximately 10% of boys and men with Klinefelter syndrome exhibit autism spectrum disorder. Therefore, Klinefelter syndrome was also excluded since our patient exhibited no clinical or social problems [8]. Finally, Cockayne syndrome (CS) is a rare, autosomal recessive disorder which was first described in 1936 by Edward Cockayne. Early descriptions of CS identified microcephaly and growth failure as cardinal clinical features. Other recognized features include hearing loss, cataracts, retinal dystrophy, and developmental delays [13, 14]. In the present case, CS was excluded since none of the patient's clinical features were consistent with this diagnosis. Thus, the present case was excluded from any associations with reported syndromes which are characterized by similar features.

The morphology of permanent premolars and molars has been reviewed extensively.

Descriptive data regarding morphological or anatomical landmarks for various types of teeth have been used to identify average variations among different populations [15, 16]. These studies provide basic knowledge for clinicians to recognize potential issues or aspects during treatment or case management. One of the anatomical landmarks which could be used as a reference for all teeth is vertical length. Despite small variations among the findings of different studies [15–19], it has generally been reported that the vertical lengths of teeth share similar averages. In the present case, an extreme variation in root length was detected in periapical radiographs (PAs) and with CBCT. Among published reports of radiculomegaly, canine teeth are most commonly affected [4, 9, 10, 20, 21]. Being able to obtain accurate root canal length measurements after treatment is also essential for determining the success of a treatment. Conventional methods for measurement involve tools such as an electronic apex locator (EAL) and PAs [22]. However, a limitation of obtaining measurements from two-dimensional PAs is that root canal length is frequently overestimated [23]. Similarly, when measuring tooth length from PAs, the likelihood of distortion relative to the projection angle of the X-ray unit must be considered. CBCT-based data can be used as a reference for evaluating root length and resorption of teeth without metal restorations in patients with malocclusions [24]. In addition, CBCT is a validated tool for exploring root canal morphology in three dimensions [25]. However, when dealing with ionizing radiation, the indication for each new image has to be critically evaluated. Particularly with regard to endodontic treatments, it must be determined whether spatial imaging is really needed or whether conventional two-dimensional PAs are sufficient, according to the ALARA principle in medical radiology [26].

For endodontic treatment of elongated roots, longer manual and rotary files are needed. Due to concerns raised nearly 40 years ago, efforts were made to standardize endodontic files and root canal filling materials. As a result, an international organization for standardization of endodontic files (ISO) was established. Currently, there are three standard lengths available for endodontic files: 21 mm, 25 mm, and 31 mm. In addition, various studies have been conducted to improve the intraoral accessibility of instruments for endodontic treatment [27]. Thus, modified endodontic files and obturation techniques have been developed (Pace et al., 2011). In some cases, KReamer veterinary instruments have been used (Vetinox; Dentsply Maillefer, Ballaigues, Switzerland). To perform obturation, a Thermafil-modified technique has been developed. Briefly, the plastic handle of a Thermafil obturator is removed and a Thermafil stem is inserted into a stainless-steel straight wire to complete the obturation. Mehran et al. described another modified technique for fabricating long files. Their technique involves cutting and removing the plastic handle of a 31-mm K-file and the handle of another file at the initiation of the flutes (thereby keeping the handle). The two parts are then attached with soldering [28]. A technique to fabricate long GuttaPercha cones has also been described by Maden et al. Briefly, an artificial canal is created in a plastic mold by using veterinary files. Thermoplastized gutta-percha is subsequently injected into the mold to create a cone [21].

Another treatment option to consider for patients with radiculomegaly involves use of Mineral Trioxide Aggregate (MTA). The introduction of bioceramics has showed promising results in the treatment of mature permanent teeth with carious exposures [29]. For example, Taha et al. reported that a full pulpotomy performed with biodentine was successful up to a year after mature permanent teeth with carious-exposed pulps, and clinical signs and symptoms indicative of irreversible pulpitis were treated [30]. In 2017, Linu et al. also reported a technique using a biometric material which exhibited better biocompatibility and sealing properties. As a result, application of this technique provided more predictable outcomes for direct pulp capping of mature permanent teeth with carious exposure [31]. Given the hypomineralization which was noted for tooth #32 in the present case, it is possible that this represents Molar-Incisor Hypomineralization (MIH), a condition which affects the enamel of permanent teeth. First permanent (adult) molars and incisors (front teeth) are most commonly affected by MIH. Normally, enamel appears white and is very hard. However, in cases of MIH, the enamel has a creamy or yellow/brown color [32]. There was no caries detected in tooth #32 in our patient.

A bony expansion was observed in the palatal sides of the patient's maxilla. This expansion was characterized as hard, bony, and painless, and it did not exhibit any signs of growth. Therefore, this expansion was considered clinically to represent a palatal exostosis. The latter is a benign lesion which can be left alone, or can be surgically removed to obtain functional benefits [33].

## 4. Conclusion

Radiculomegaly has been associated with many syndromes in the literature, while only a few reports of nonsyndromic cases have been published. Here, we report a nonsyndromic case of generalized radiculomegaly in a Saudi individual. Initially, patients with radiculomegaly should receive counseling regarding caries prevention since extensive caries can lead to pulpal involvement. Furthermore, the latter may require root canal treatment or dental extractions, and these have adverse treatment complications to consider. If it is decided that endodontic treatment is needed, special management protocols and tools should then be considered.

## Figures and Tables

**Table 1:** Published cases of nonsyndromic radiculomegaly.

Title of publication	Authors	Year of publication	Journal
Cuspid gigantism	Hayward JR	1980	Oral Surgery, Oral Medicine, Oral Pathology
A very long cuspid	Weine FS	1986	Journal of Endodontics
Radiculomegaly of canines and congenital cataracts – a syndrome?	Marashi and Gorlin	1990	Oral Surgery, Oral Medicine, Oral Pathology
A very large maxillary cuspid	Wilkie and Chambers	1990	Oral Surgery, Oral Medicine, Oral Pathology
Long root of deciduous anterior teeth	Fujmura et al.	2008	Odontology
Bilateral second premolars agenesia together with a unilateral canine radiculomegaly	Kemoli and Junior	2017	Contemporary Clinical Dentistry
Radiculomegaly: a case report of this rare dental finding with review of the associated oculofacio-cardio-dental syndrome	Smith et al.	2018	Oral and Maxillofacial Pathology

## References

[1] Huang X.-F., Chai Y. (2012). Molecular regulatory mechanism of tooth root development. *International Journal of Oral Science*.

[2] Luder H. U. (2015). Malformations of the tooth root in humans. *Frontiers in Physiology*.

[3] Suda N., Moriyama K. (2009). Human diseases associated with abnormal tooth roots. *Journal of Oral Biosciences*.

[4] Kato J., Kushima K., Kushima F. (2018). New radiological findings and radiculomegaly in oculofaciocardiodental syndrome with a novel BCOR mutation: a case report. *Medicine*.

[5] Hayward J. R. (1980). Cuspid gigantism. *Oral Surgery, Oral Medicine, and Oral Pathology*.

[6] Weine F. S. (1986). A very long cuspid!. *Journal of Endodontia*.

[7] Wilkie G. J., Chambers I. G. (1990). A very large maxillary cuspid. *Oral Surgery, Oral Medicine, and Oral Pathology*.

[8] Lähdesmäki R., Alvesalo L. (2007). Root lengths in the permanent teeth of Klinefelter (47,XXY) men. *Archives of Oral Biology*.

[9] Kemoli A. M., Junior T. M. (2017). Bilateral second premolars agenesia together with a unilateral canine radiculomegaly. *Contemporary Clinical Dentistry*.

[10] Smith M. H., Cohen D. M., Bhattacharyya I., Islam N. M., Kashtwari D. (2018). Radiculomegaly: a case report of this rare dental finding with review of the associated oculo-facio-cardio-dental syndrome. *Oral Surgery, Oral Medicine, Oral Pathology, Oral Radiology*.

[11] Marashi A. H., Gorlin R. J. (1992). Radiculomegaly of canine teeth and congenital cataracts: confirmation of a syndrome. *American Journal of Medical Genetics*.

[12] Davoody A., Chen I.-P., Nanda R., Uribe F., Reichenberger E. J. (2012). Oculofaciocardiodental syndrome: a rare case and review of the literature. *The Cleft Palate-Craniofacial Journal*.

[13] Bloch-Zupan A., Rousseaux M., Laugel V. (2013). A possible cranio-oro-facial phenotype in Cockayne syndrome. *Orphanet Journal of Rare Diseases*.

[14] Wilson B. T., Stark Z., Sutton R. E. (2016). The Cockayne Syndrome Natural History (CoSyNH) study: clinical findings in 102 individuals and recommendations for care. *Genetics in Medicine*.

[15] Carrotte P. (2004). Endodontics: Part 4 Morphology of the root canal system. *British Dental Journal*.

[16] Nelson S. J., Ash M. M., Ash M. M. (2010). *Wheeler’s Dental Anatomy, Physiology, and Occlusion*.

[17] Jain A., Bahuguna R. (2011). Root canal morphology of mandibular first premolar in a gujarati population - an in vitro study. *Dental Research Journal*.

[18] Pécora J. D., Saquy P. C., Sousa Neto M. D., Woelfel J. B. (1992). Root form and canal anatomy of maxillary first premolars. *Brazilian Dental Journal*.

[19] Pécora J. D., Woelfel J. B., Sousa Neto M. D. (1991). Morphologic study of the maxillary molars. 1. External anatomy. *Brazilian Dental Journal*.

[20] Iwase M., Nishijima H., Kondo G., Ito M. (2015). Radiculomegaly of permanent canines and first premolars: report of two cases in conjunction with oculo-facio-cardio-dental syndrome. *International Journal of Case Reports and Images*.

[21] Maden M., Savgat A., Görgül G. (2010). Radiculomegaly of permanent canines: report of endodontic treatment in OFCD syndrome. *International Endodontic Journal*.

[22] Jeger F. B., Janner S. F. M., Bornstein M. M., Lussi A. (2012). Endodontic working length measurement with preexisting cone-beam computed tomography scanning: a prospective, controlled clinical study. *Journal of Endodontia*.

[23] Williams C. B., Joyce A. P., Roberts S. (2006). A comparison between in vivo radiographic working length determination and measurement after extraction. *Journal of Endodontia*.

[24] Kim S.-Y., Lim S.-H., Gang S.-N., Kim H.-J. (2013). Crown and root lengths of incisors, canines, and premolars measured by cone-beam computed tomography in patients with malocclusions. *The Korean Journal of Orthodontics*.

[25] Michetti J., Maret D., Mallet J.-P., Diemer F. (2010). Validation of cone beam computed tomography as a tool to explore root canal anatomy. *Journal of Endodontics*.

[26] Pauwels R., Beinsberger J., Collaert B. (2012). Effective dose range for dental cone beam computed tomography scanners. *European Journal of Radiology*.

[27] Kim J.-Y., Lee S.-H., Lee G.-H., Park S.-H. (2010). Study of endodontic working length of Korean posterior teeth. *Journal of Korean Academy of Conservative Dentistry*.

[28] Taramsari M., Dalili Kajan Z., Atash Biz Yeganeh L. (2017). Case report: an oculofaciocardiodental syndrome: challenges in endodontic treatment. *Journal of Dentomaxillofacial*.

[29] Taha N. A., Ahmad M. B., Ghanim A. (2017). Assessment of mineral trioxide aggregate pulpotomy in mature permanent teeth with carious exposures. *International Endodontic Journal*.

[30] Taha N. A., Abdelkhader S. Z. (2018). Outcome of full pulpotomy using Biodentine in adult patients with symptoms indicative of irreversible pulpitis. *International Endodontic Journal*.

[31] Linu S., Lekshmi M. S., Varunkumar V. S., Sam Joseph V. G. (2017). Treatment outcome following direct pulp capping using bioceramic materials in mature permanent teeth with carious exposure: a pilot retrospective study. *Journal of Endodontics*.

[32] Almuallem Z., Busuttil-Naudi A. (2018). Molar incisor hypomineralisation (MIH) – an overview. *British Dental Journal*.

[33] Isha M., Nimma Vijayalaxmi B., Ramaswami E., Desai Jimit J. (2018). Bony exostoses: case series and review of literature. *Acta Scientific Dental Sciences*.

